# Group Testing-Based Robust Algorithm for Diagnosis of COVID-19

**DOI:** 10.3390/diagnostics10060396

**Published:** 2020-06-11

**Authors:** Jin-Taek Seong

**Affiliations:** Department of Convergence Software, Mokpo National University, Muan 58554, Korea; jtseong@mokpo.ac.kr

**Keywords:** COVID-19, diagnosis, group testing, posterior probability, robust algorithm

## Abstract

At the time of writing, the COVID-19 infection is spreading rapidly. Currently, there is no vaccine or treatment, and researchers around the world are attempting to fight the infection. In this paper, we consider a diagnosis method for COVID-19, which is characterized by a very rapid rate of infection and is widespread. A possible method for avoiding severe infections is to stop the spread of the infection in advance by the prompt and accurate diagnosis of COVID-19. To this end, we exploit a group testing (GT) scheme, which is used to find a small set of confirmed cases out of a large population. For the accurate detection of false positives and negatives, we propose a robust algorithm (RA) based on the maximum a posteriori probability (MAP). The key idea of the proposed RA is to exploit iterative detection to propagate beliefs to neighbor nodes by exchanging marginal probabilities between input and output nodes. As a result, we show that our proposed RA provides the benefit of being robust against noise in the GT schemes. In addition, we demonstrate the performance of our proposal with a number of tests and successfully find a set of infected samples in both noiseless and noisy GT schemes with different COVID-19 incidence rates.

## 1. Introduction

**Figure 1:**
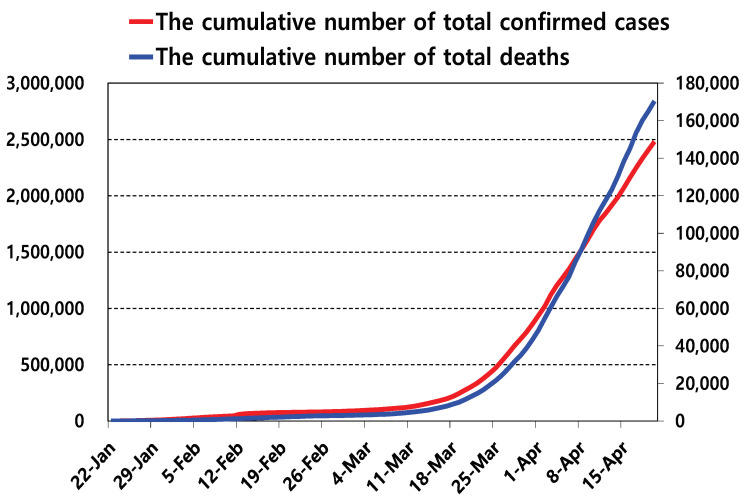
The cumulative number of confirmed cases and the cumulative number of deaths by COVID-19 by 20 April [13].

The ability to test for COVID-19, which has been characterized as a rapid contagion, is still insufficient to meet global health needs. COVID-19 transmission occurs between individuals, becoming a greater threat when using public facilities such as hospitals, religious facilities, schools, military units, and cruise ships. COVID-19 causes diseases such as pneumonia and acute respiratory distress syndrome (ARDS), which have low mortality rates but can lead to death. Clinical and physical symptoms may include shortness of breath, fever, cough, anosmia and gastrointestinal symptoms. COVID-19 is characterized by a low mortality rate but a very contagious nature. By April 20 in 2020, the number of confirmed COVID-19 cases was over 2.8 million and more than 148,000 people have died, as shown in Figure 1. In the future, the number of infected people is expected to continue to increase across developing countries.

In the most recent papers published online [1,2,3], group testing (GT) has been used to provide a quick and efficient solution to test the methods used to screen for COVID-19 infected people. The number of infected people is currently increasing by hundreds of thousands of people per day; thus, instead of individual testing, GT has shown to be economical and efficient, as it can reduce the number of required tests for COVID-19. GT is not a recently proposed approach; it was first proposed by Dorfman in 1943. [4]. To date, GT has been exploited in a wide range of applications in biology [5], communication theory [6], computer science [7], and mathematics [8]. The use of fundamental GTs extends to error correction codes [9], identifying available multiple access channels [10], detecting malicious attacks in security networks [11], testing the quality of products [12], and many others.

Next, a brief introduction to GT and an important testing method are discussed. GT began with a project to find all soldiers infected with syphilis by the U.S. health service during World War II [4]. Syphilis testing took blood cases from individual soldiers to test for syphilis infection. However, as the number of soldiers tested for syphilis was very large, the cost of the testing was high and a great deal of time was required to find a new test method [5]. Consequently, this motivated the development of the GT framework [4].

In conventional GT, syphilis testing was performed using the following method. First, blood cases from several soldiers were mixed into a single pool to see whether they react to the syphilis test. When the result was positive, it meant that at least one soldier was infected with syphilis. On the other hand, in the case of a negative, it could be confirmed that all blood cases used for the syphilis testing were not infected with syphilis. Thus, it can be stated that most soldiers were not infected with syphilis, and only a handful of soldiers had syphilis. The main challenges of GT are as follows: first, determining the samples to be included in a pool; second, a detection algorithm must be used to find a set of infected people out of the large number of samples.

Briefly, the GT problem is clearly defined as follows: Let *T* be the number of tests required to find a set of confirmed cases when *D* people of the total population *N* are infected, and Let A be the group matrix with *T* rows and *N* columns. The role of the group matrix is to map the samples to be grouped in the test. For i∈{1,2,…,T} and j∈{1,2,…,N}, if the *i*th group includes the *j*th sample, the corresponding entry $A_{ij}$ of the group matrix *A* is represented as $A_{ij}=1$; otherwise, we express this as $A_{ij}=0$. In other words, when the entry of the group matrix is 1, GT is performed including the *j*th sample indicating the corresponding column. And we define the following terminologies and notations: bold upper and lower case letters denote matrices and column vectors, calligraphic letters denote sets, and Pr() is a probability.

Some recent works [1,2,3] have proposed GT methods or the rapid diagnosis of COVID-19. However, these works had the disadvantage of not being able to accurately pinpoint the outcomes of tests when there are false negatives and false positives. To overcome this inaccuracy, in this paper, we propose a robust algorithm (RA) (this term does not refer to a class of GT algorithms, it refers the meaning of robust decoding for GT problems with false negative and false positives) and demonstrate its performance against noise, even if errors occur in the output results. In order to detect the small number of confirmed cases of COVID-19, we exploit the detection algorithm using the maximum a posteriori probability (MAP), and we see that the proposed RA provides the benefit of being robust against noise. In addition, we show how many tests are needed, depending on the incidence rate of COVID-19. Furthermore, we demonstrate the robustness of our proposed algorithm to noise, as compared to other algorithms.

This paper is organized as follows. In Section 2, we investigate the related works in detection algorithms used for GT. The challenges of GT are defined, in detail, in Section 3. The description of the detection algorithm proposed in this paper is provided in Section 4, and we show the simulation results and compare them with other results. Finally, in Section 5, we conclude by showing that we have obtained meaningful results and findings.

## 2. Related Works

A number of detection algorithms for GT problems have been proposed since the detection algorithm was first introduced by Dorfman. This section aims to review some detection algorithms related to GT.

The detection algorithm to be reviewed first is the binary splitting algorithm [5]. This algorithm is generally called the optimal adaptive algorithm in GT. The binary splitting algorithm is used to find less than or equal to *D* infected cases in *N* cases, and is summarized, as follows, according to the size of *N* and *D*: In the initial step, in the case where N≤2D−2, finding *D* confirmed cases is performed by individual testing. This means that individual testing is better than GT when there are many confirmed cases. Otherwise, set L=N−D+1 and $\alpha :=\lfloor \log_{2}L/D\rfloor$, respectively. In the next step, GT is performed by a set of cases with a size of $2^{\alpha }$ for every testing. Here, when the result of this GT is negative, all cases in this pool are determined to be normal. Then, the cases are reset with a size of $N=N-2^{\alpha }$ and GT is performed in the same manner as in the first step. On the other hand, using a binary search, one infected sample and the other normal cases *S* are again set as follows: N=N−S−1 and D=D−1.

The number of tests *T* for the generalized binary splitting (GBS) algorithm with respect to p>0, *N*, and *D* is required to be $T=D\left(\alpha +2\right)+p-1$ where, in the case of high N/D, *T* converges to $D\log_{2}\left(N/D\right)$ [5]. In a recent paper [1], the authors analyzed the performance of the binary splitting algorithm for COVID-19. Their results showed very close optimal results for the lower bounds obtained from information–theoretic bounds [1].

Next, we review the COMP (Combinatorial Orthogonal Matching Pursuit) algorithm [14]. This algorithm is a class of non-adaptive GT algorithms. The COMP algorithm works as follows: first, each entry of the group matrix is assumed to follow an i.i.d. (independent and identically distributed) Bernoulli probability distribution with the probability 1/D for 1, and 1−1/D for 0. The key idea of the COMP algorithm is to combine the columns of the group matrix corresponding to the individual cases participating in a pool. As with the conventional GT problems, the results are determined to be positive or negative depending on the existence of confirmed cases. The number of tests *T* for the COMP algorithm with any constant β>0 and where the average error probability is less than or equal to $N^{-\beta }$ is as follows: $T\geq eD\left(1+\beta \right)\ln N$ [14].

Another algorithm, which is an extended version of the COMP algorithm, is called Definite Defectives (DD), which is used to remove false positive errors [15]. The performance of the DD algorithm improves on that of the COMP algorithm, where the detection method of the DD algorithm exploits useful attributes of the COMP algorithm. Note that the normal cases obtained from the COMP algorithm are surely detected without false negative error. Therefore, the DD algorithm only generates false negatives compared to the COMP algorithm.

The SCOMP (Sequential COMP) algorithm takes advantage of the fact that the DD algorithm does not cause errors until the last step [15]. All remaining cases are assumed to be normal. Let K be the set of cases detected to be infected; if the test contains at least one infected sample from the set K, a positive result is obtained. Note that it cannot be said that the set of confirmed cases detected by the DD algorithm includes all positive results. This means that test results that cannot be clearly identified have to contain one hidden defect sample. Simulation results using the SCOMP algorithm have shown results close to the optimal ones [15]. The other results of adaptive and noisy GT problems are presented in [16,17,18].

## 3. Group Testing for Diagnosis of COVID-19

In this section, we define the GT framework in detail. Let $x={\left(x_{1},x_{2},\ldots ,x_{N}\right)}^{T}$ be a binary input vector with size *N*. If the *j*th entry of x is infected, then we write $x_{j}=1$. Otherwise, $x_{j}=0$. Thus, all the entries of the input vector x are represented in binary form. In this paper, we assume that all the entries of x are independent and identically distributed (i.i.d.) following the Bernoulli distribution with the incidence rate ϵ for COVID-19,

(1) $$\mathrm{Pr}\left(x_{j}\right)=\left\{\begin{array}{cc} 1-\epsilon & \;\;\mathrm{for}\;\;x_{j}=0,\\ \epsilon & \;\;\mathrm{for}\;\;x_{j}=1, \end{array}\right.$$

where $\epsilon =\frac{D}{N}$ denotes the incidence rate for COVID-19. Indeed, the incidence rate of COVID-19 is assumed to be a very small value.

The following describes the mathematical expression of GT in more detail. Each entry $y_{i}$ of the output result vector y is expressed as follows by using the input vector x and the group matrix A:

(2) $$y_{i}=\left(\overset{N}{\underset{j=1}{⋁}}\left(A_{ij}\wedge x_{j}\right)\right)⊕z_{i},$$

where $y_{i}$ is the output of the GT scheme, assuming that the *i*th additive noise $z_{i}$ is used. In the case where there is no noise, the output $z_{i}$ of the *i*th group is positive if there is at least one infected person in this group, which is indicated by $y_{i}=1$; otherwise, it is 0. We express the output of the GT, as a vector, as follows: $y={\left(y_{1},y_{2},\ldots ,y_{T}\right)}^{T}$. The symbols ∨, ∧, and ⊕ denote the logical AND, OR, and XOR operations, respectively. Let $z_{i}$ be the additive noise in the *i*th group leading to false positives and negatives for the pure output result. It is assumed that the noise $z_{i}$ has the following probability distribution:

(3) $$\mathrm{Pr}\left(z_{i}\right)=\left\{\begin{array}{cc} 1-\sigma & \;\;\mathrm{for}\;\;z_{i}=0,\\ \sigma & \;\;\mathrm{for}\;\;z_{i}=1. \end{array}\right.$$

In fact, the probabilities of false positive and negative errors are not the same. For example, Knill et al. in [19] showed that the false positive and negative rates were up to 13% and 5% through actual experiments, respectively. In addition, the Z-channel model with only one error (e.g., false positive or negative) was considered in [18]. In this paper, we consider the symmetric noise model as shown in Equation (3). Then, we obtain all the entries of the output vector y of the GT scheme. This mathematical expression of the GT scheme takes advantage of the easy handling of the states of x, A, and y in our proposed algorithm.

**Figure 2:**
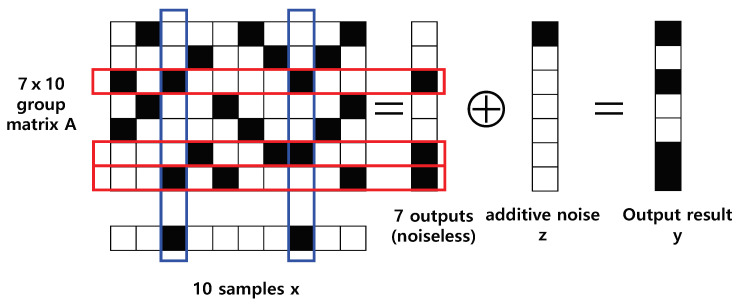
One example of the simple group testing (GT) scheme with a 7×10 group matrix A, 10 samples x, additive noise z with one error, and seven output results y. The black and white boxes indicate 1 and 0, respectively. The third and eighth samples are included in the third group test, and its output is 1. The outputs of the sixth and seventh tests are 1. The first pure output is 0; however, the additive noise is included, and the corresponding output is flipped.

Figure 2 shows an example of the GT scheme with a 7×10 group matrix A, 10 samples x, additive noise z with one error, and 7 output results y. The white and black cells represent 0 and 1, respectively. The blue box represents the groups in which the infected samples participate, and the red box shows the results of the GT. In this example, the third and eighth samples participate in the third group test, and its output is 1. In addition, the outputs of the sixth and seventh tests are 1. The first pure output is 0; however, the additive noise is included and the corresponding output is flipped.

## 4. Detection of Confirmed Cases of COVID-19

### 4.1. Proposed Robust Algorithm

In this section, we propose a robust algorithm (RA) for GT. This RA is based on the use of MAP. Note that the problem of finding the optimal MAP solution in GT is NP-hard. Although it is difficult to find the optimal solution for this argument, many researchers have tried to find sub-optimal approaches, which are close to the optimal one. Among them, the performance of the belief propagation algorithm, introduced by Mackey in [20], showed results close to the Shannon limits in channel coding theory.

To handle our proposed RA, we assume the following: each person $x_{j}$ has a prior probability of having an infected and normal state (given by Equation (1)) under the GT scheme, assuming that the input vector, group matrix, and output result are mutually independent. The challenge for GT is finding the MAP combination of the estimated $\hat{x}$ cases, given the observed output y. This is formulated as

(4) $$\begin{array}{cc} \hat{x} & =\arg\max\mathrm{Pr}\left(x|y\right)\\ \; & =\arg\max_{x_{j}\in \left\{0,1\right\}}\prod_{j=1}^{N}\mathrm{Pr}\left(x_{j}|y\right), \end{array}$$

where the second equality comes from the independence assumption of the prior cases.

Using Bayes’ rule, the conditional probability $\mathrm{Pr}\left(x_{j}|y\right)$ can be rewritten as

(5) $$\begin{array}{cc} \mathrm{Pr}\left(x_{j}|y\right) & =\sum_{x\backslash x_{j}}\mathrm{Pr}\left(x|y\right)\\ \; & \propto \sum_{x\backslash x_{j}}\mathrm{Pr}\left(y|x\right)\mathrm{Pr}\left(x\right)\\ \; & =\sum_{x\backslash x_{j}}\prod_{i=1}^{T}\mathrm{Pr}\left(y_{i}|x\right)\prod_{j=1}^{N}\mathrm{Pr}\left(x_{j}\right), \end{array}$$

where the notation $\sum_{x\backslash x_{j}}$ denotes a summation over all entries of x except the entry $x_{j}$, the second proportional relation holds due to Bayes’ rule and the last equality comes from the independent assumption. The aim of the proposed algorithm is to find the maximized marginal probability for each sample in Equation (5).

**Figure 3:**
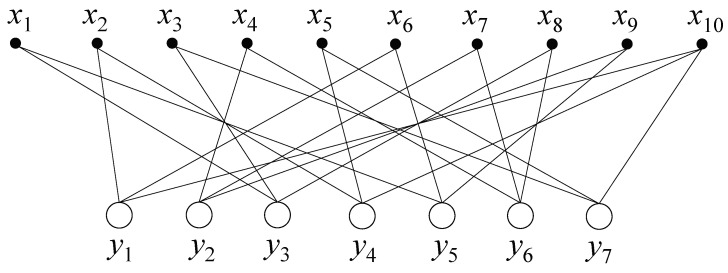
Graphical representation with 10 samples and seven output results for the example of the group testing (GT) scheme shown in Figure 2.

Next, we describe the key idea of the RA proposed in this paper. Before describing our algorithm, the graphical representation of one example of the GT scheme is represented in Figure 3. There are 10 samples—$x_{1}$ through $x_{10}$—and seven output results—$y_{1}$ through $y_{7}$. As the first row of *A*, as shown in Figure 2, is [0 1 0 0 0 1 0 0 0 1], there are three edges between the first output $y_{1}$ and three samples, $x_{2}$, $x_{6}$ and $x_{10}$, in Figure 3. In the same way, other edges between the samples and the outputs can be drawn as shown in Figure 3.

Let $L\left(i\right)=\left\{x_{j}:A_{ij}=1\right\}$ be the set of samples participating in the *i*th group, and $V\left(j\right)=\left\{y_{i}:A_{ij}=1\right\}$ be the set of groups participating in the *j*th sample. We also use L(i)\{j} to denote the set L(i) excluded the *j*th sample, and V(j)\{i} to denote the set V(j) excluding the *i*th group. The RA proposed in this paper is mainly described as a process in which two marginal probabilities exchange information in each iteration. Note that we aim to find the maximum posterior probability for each sample, as in the last line of Equation (5). In other words, the two conditional probabilities $\mathrm{Pr}(x_{j}|y)$ and $\mathrm{Pr}(y_{i}|x)$ are exchanged with each other to maximize the posterior probability. Let $\xi_{ji}\propto \mathrm{Pr}\left(x_{j}|y\right)$ be the downward message from sample $x_{j}$ to output $y_{i}$, and $\delta_{ij}\propto \mathrm{Pr}\left(y_{i}|x\right)$ be the upward message from output $y_{i}$ to sample $x_{j}$. Both messages are expressed as conditional probabilities. As mentioned in Equation (5), the two messages exchange their information with each other. The downward message $\xi_{ji}$ is a function of the upward message $\delta_{ij}$, and vice versa. In the example of Figure 3, the downward message $\xi_{x_{10}y_{1}}$ is obtained from the two upward messages $\delta_{y_{4}x_{10}}$ and $\delta_{y_{7}x_{10}}$. Conversely, the upward message $\delta_{y_{1}x_{10}}$ is obtained from the two downward messages $\xi_{x_{2}y_{1}}$ and $\xi_{x_{6}y_{1}}$.

Now, the RA updates the messages $\xi_{ji}$ and $\delta_{ij}$ associated with each edge between the sample $x_{j}$ and the output $y_{i}$. There are three steps to estimate each input sample: initialization, updating the messages $\xi_{ji}$ and $\delta_{ij}$, and tentative decoding to check the constraint condition in Equation (2). In the initialization step, we define the probability distribution of x in Equation (1), generate the group matrix A with a random design (i.e., low-density parity check (LDPC) codes, as in [20]), and obtain the output result y from the given A and x. We aim to find an (unknown) input vector x by using (known) A and y. In addition, the initial downward message $\xi_{ji}$ can be obtained from the prior probability distribution of Equation (1), assuming that the upward messages for 0 and 1 are equally distributed.

Next, we consider the upward message $\delta_{ij}$ from output $y_{i}$ to sample $x_{j}$. This message $\delta_{ij}$ is obtained as follows:

(6) $$\delta_{ij}=\sum_{{⋁}_{j=1}^{N}\left(A_{ij}\wedge x_{j}\right)}\prod_{j^{\prime }\in L\left(i\right)\backslash \left\{j\right\}}\xi_{j^{\prime }i},\mathrm{Pr}\left(z_{i}\right)$$

where the constraint condition in Equation (2) is satisfied as ${⋁}_{j=1}^{N}\left(A_{ij}\wedge x_{j}\right)$ in the noiseless GT scheme. The summation of Equation (6) is used to collect all the associated downward probabilities that satisfy the constraint condition of Equation (2). To update the upward message, the noisy probability $\mathrm{Pr}(z_{i})$ is multiplied.

The downward message $\xi_{ji}$ can be written as

(7) $$\xi_{ji}=\lambda \mathrm{Pr}\left(x_{j}\right)\prod_{i^{\prime }\in V\left(j\right)\backslash \left\{i\right\}}\delta_{i^{\prime }j},$$

where the variable λ is used for normalization of the total probability. Let $\mathrm{Pr}\left({\hat{x}}_{j}\right):=\mathrm{Pr}\left(x_{j}|y\right)$ be the posterior probability for the sample $x_{j}$. Finally, we determine a maximum probability for 0 or 1, as defined in Equation (5),

(8) $${\hat{x}}_{j}=\arg\max_{x_{j}\in \left\{0,1\right\}}\mathrm{Pr}\left(x_{j}\right)\prod_{i\in V(j)}\delta_{ij}.$$

Using Equations (6) and (7), the proposed RA iteratively updates the messages $\xi_{ji}$ and $\delta_{ij}$; that is, while the algorithm is running, the posterior probability of each sample moves to converge in general. Even when the algorithm has been operated for the maximum number of iterations, the posterior probability may not converge. In this case, we assume that the sample is unreliable and perform individual testing instead of GT. In the final step, we perform individual testing by picking only unstable samples. Algorithm 1 describes our proposed RA for the detection of infected people in COVID-19.
**Algorithm 1:** Proposed Robust Algorithm (RA).
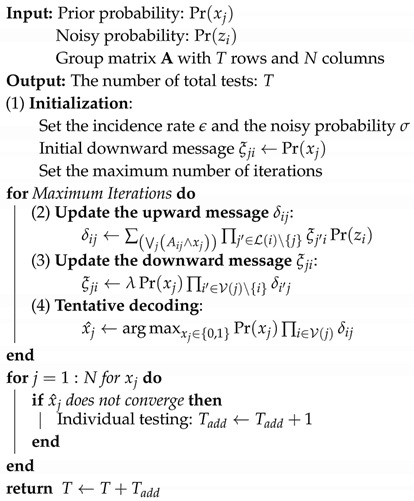


Let $T_{add}$ be the number of samples that requires individual testing when the posterior probability does not converge. The total number of tests *T* for successfully finding infected people in COVID-19 is obtained as follows:

(9) $$T=T+T_{add}.$$

### 4.2. Simulation Results

**Figure 4:**
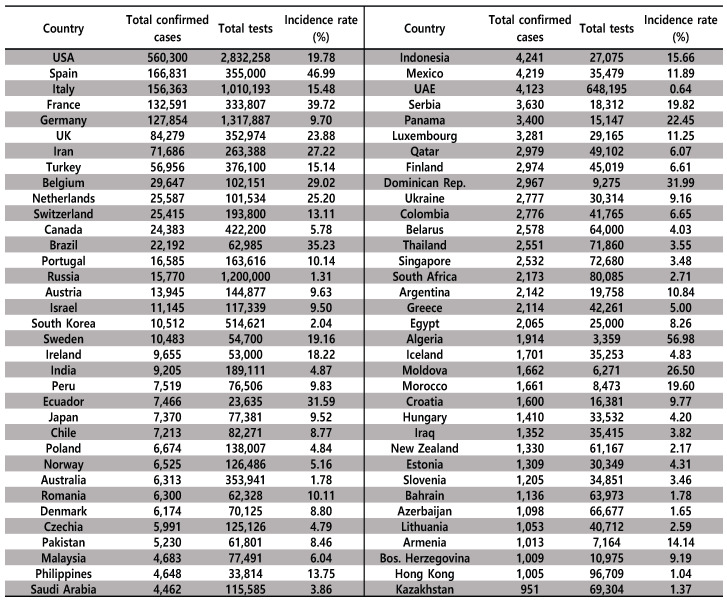
The total number of confirmed cases and tests for COVID-19 in each country as of 12 April 2020 [13]. The average incidence rate is 12.89%, with a total number of confirmed cases of 1,743,883 and a total number of tests of 13,531,095.

COVID-19 has a different incidence rate in each country. Figure 4 shows the number of infected people and number of tests in each country. The statistics shown in Figure 4 list the countries with the largest number of confirmed cases as of April 12, 2020. China, the first affected country, was excluded from Figure 4, due to a lack of information on the number of tests. In addition, the average incidence rate for the most-affected countries, as shown in Figure 4, is 12.89%, where the total number of confirmed cases is 1,743,883 and the total number of tests is 13,531,095. However, this incidence rate is reported to be lower than the actual case, as it is not recommended to actively test suspected patients. We consider South Korea as our simulation environment for the diagnosis of COVID-19; the reason for this is that it adopted the world’s most objective and aggressive countermeasures to COVID-19. According to these results, the incidence rate of COVID-19 in South Korea is very low, at about 2%.

We implemented a decoding algorithm for the GT scheme to find people with infected COVID-19 in noiseless (σ=0) and both false positive and negative settings (σ≠0). Our proposed algorithm is based on the belief propagation algorithm of LDPC codes [20] in channel coding theory, which has shown performance close to the Shannon limit in information theory. The difference between Mackey’s method [20] and the proposed RA is revealed in the operations. In the case of channel coding, the operation is performed over Finite Fields; however, GT uses Boolean operations such as AND, OR, and XOR. We evaluate the performance on RAs for the GT schemes. To this end, we set the simulation environment as follows: the defective samples are generated from the probability distribution in Equation (1) with different incidence rates ϵ, and the group matrix comes from LDPC codes with five constant weights in each column. Additionally, we set the number of maximum iterations as 20 for the RA. Throughout this paper, we consider N=1000, assuming that the diagnosis of COVID-19 is carried out 1000 times simultaneously on one site. All the results for the number of tests are averaged from 100 experiments. The computational complexity of the RA based on the belief propagation algorithm in LDPC codes is O(NlogN) in [20]. In addition, the relationship between the number of iterations and the performance on the belief propagation algorithm by using the analysis of density evolution under binary erasure and symmetry channels was presented in [21]. Intuitively, the greater the number of iterations of belief propagation decoding, the better the performance, but in order to achieve such a conclusion, it is necessary to have characteristics regarding the generation of a group matrix (e.g., stopping set) in [21].

Next, we evaluate the total number of tests *T* to successfully find infected people with different incidence rates in the noiseless GT scheme, as shown in Table 1. First, the information-theoretic bound was obtained from [22], which is exploited (by Fano’s inequality [23]) as a lower bound on the number of tests. Dorfman’s method [4], the DT method [1], and the GBS method [5] were evaluated at N=1000 and different incidence rates of ϵ (0.01, 0.02, 0.03, 0.04, 0.05, and 0.1), whose values were based on the statistics of the infected people for COVID-19 as of 12 April 2020, as shown in Figure 4. All the statistical results for the number of tests *T* are shown in Table 1. In the noiseless schemes, the best method for the detection of COVID-19 was the GBS algorithm [5], the results of which were close to the information-theoretic bound. This method showed the best performance but has the inconvenience of not being able to test all samples simultaneously. In contrast, our proposed RA offers the advantage of GT all the samples at once using a predefined group matrix. In other words, in the GBS method, the current test is determined based on the results of the previous test, whereas the RA processes all tests at the same time, so it can be inspected in large quantities.

**Figure 5:**
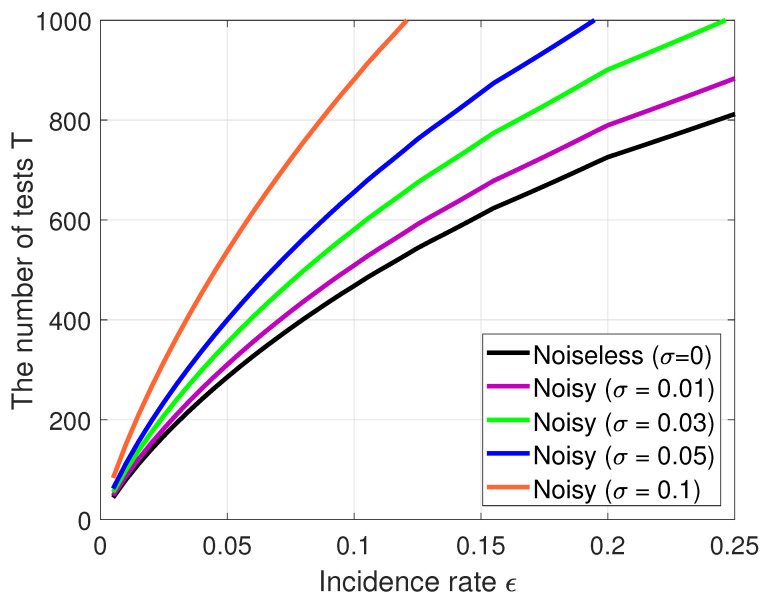
Comparisons of the number of tests *T* on information-theoretic bounds in the noisy GT schemes with a size of N=1000, different incidence rates ϵ (0.001–0.25), and noise levels σ (0.01, 0.03, 0.05, and 0.1).

The main advantage of our algorithm, compared to other algorithms, is that it is noise-resistant. Other detection methods shown in Table 1 have better performance in the noiseless GT. However, there exists noise in the GT framework, so it is limited when using these methods. We need a way to find infected samples even when the GT is noisy. To this end, we consider the noisy GT scheme with different noise values σ, which is formulated by Equation (3) as the false positive and the false negative of the GT. Figure 5 shows how many total tests on the theoretical bounds [22] are required to successfully find different incidence rates ϵ in the noisy GT schemes with N=1000, where σ=0.01, 0.03, 0.05, and 0.1. As shown in Figure 5, if the incidence rate ϵ is greater than 0.2 and the noise probability σ is greater than 0.05, individual testing is better that the GT scheme. In other words, the GT scheme in COVID-19 is suitable for the reduction of the number of tests when the incidence rate is less than 0.2, when assuming σ=0.05. Table 2 shows the performance for the number of tests *T* of our proposed RA method for the noisy GT scheme, in which there are different incidence rates ϵ and noise probabilities σ. Note that the interpretation of the false negative is more complicated because there may be contaminated input samples. In addition, test results including a large number of defective samples are unlikely to lead to false negatives, even with noise. In [24], authors showed that it is easier to find false negatives than positive ones. It also led to improved results on the number of tests in the case of false negatives.

The reason for this robustness against noise is as follows: first, the RA updates all the posterior probabilities of the unknown binary input vector at every iteration, where it comes from exchanging upward and downward messages with each other. It is used to find the uncorrupted value using the beliefs of neighboring nodes even if the test result changes due to false negative or positive errors. As the number of iterations of the algorithm increases, the posterior probability gradually converges once there is cycle-free in the bipartite graph of the GT scheme with the low noise. Note that generating a group matrix with cycle-free is difficult in the large length. In our proposed RA, as the noise increases, the belief propagation does not work, so it falls into a region where errors cannot be corrected. For more details of effect of noise for belief propagation algorithms that are robust to noise, please refer to Lav’s paper in [25].

## 5. Conclusions

In this paper, we considered a diagnosis method for COVID-19, which has been characterized by a very rapid rate of infection and is widespread. A possible method for avoiding severe infections is to stop the spread of the infection in advance by the prompt and accurate diagnosis of COVID-19. To this end, we exploit a group testing (GT) scheme, which is used to find a small set of confirmed cases out of a large population. To this end, results using GT as a diagnostic method for COVID-19 were presented. For the detection of false positives and negatives, we proposed an RA based on the MAP. The core idea of RA is that it exploits iterative detection to propagate beliefs to neighbor nodes by exchanging marginal probabilities between input and output nodes. As a result, we demonstrated that the our proposed RA provides the benefit of being robust in the GT schemes against noise when false positive and false negative outputs occur. In addition, through a number of tests, we showed the ability of our proposed method to successfully find a set of infected people in noiseless and noisy GT schemes with different incidence rates of COVID-19.

## Figures and Tables

**Table 1:** Comparisons of the total number of tests *T* expressed as mean to successfully find infected people with different incidence rates in the noiseless GT scheme, where N=1000 and σ=0.

Incidence Rate ϵ	0.01	0.02	0.03	0.04	0.05	0.1
Lower bound [22]	80	141	194	242	286	469
Dorfman’s method [4]	196	274	335	384	432	594
Divide and Test method [1]	81	144	198	275	289	477
GBS method [5]	88	153	209	258	305	494
Our proposed method	108	168	231	306	377	593

**Table 2:** Comparisons of the total number of tests *T* expressed as (mean, standard deviation) to successfully find infected people with different incidence rates in the noisy GT scheme, where N=1000 and σ=0.01, 0.03, 0.05.

	Incidence Rate ϵ	0.01	0.02	0.03	0.04	0.05	0.1
σ=0.01	Lower bound [22]	(88, 0.0)	(154, 0.0)	(212, 0.0)	(264, 0.0)	(312, 0.0)	(521, 0.0)
	Our proposed method	(119, 5.3)	(184, 8.9)	(253, 10.6)	(334, 11.1)	(417, 11.0)	(658, 11.8)
σ=0.03	Lower bound [22]	(100, 0.0)	(175, 0.0)	(241, 0.0)	(301, 0.0)	(355, 0.0)	(581, 0.0)
	Our proposed method	(136, 5.3)	(210, 9.1)	(289, 10.1)	(383, 11.2)	(476, 11.5)	(749, 11.9)
σ=0.05	Lower bound [22]	(113, 0.0)	(198, 0.0)	(272, 0.0)	(340, 0.0)	(406, 0.0)	(658, 0.0)
	Our proposed method	(153, 5.8)	(237, 9.2)	(325, 10.7)	(431, 11.9)	(539, 12.1)	(846, 12.4)
